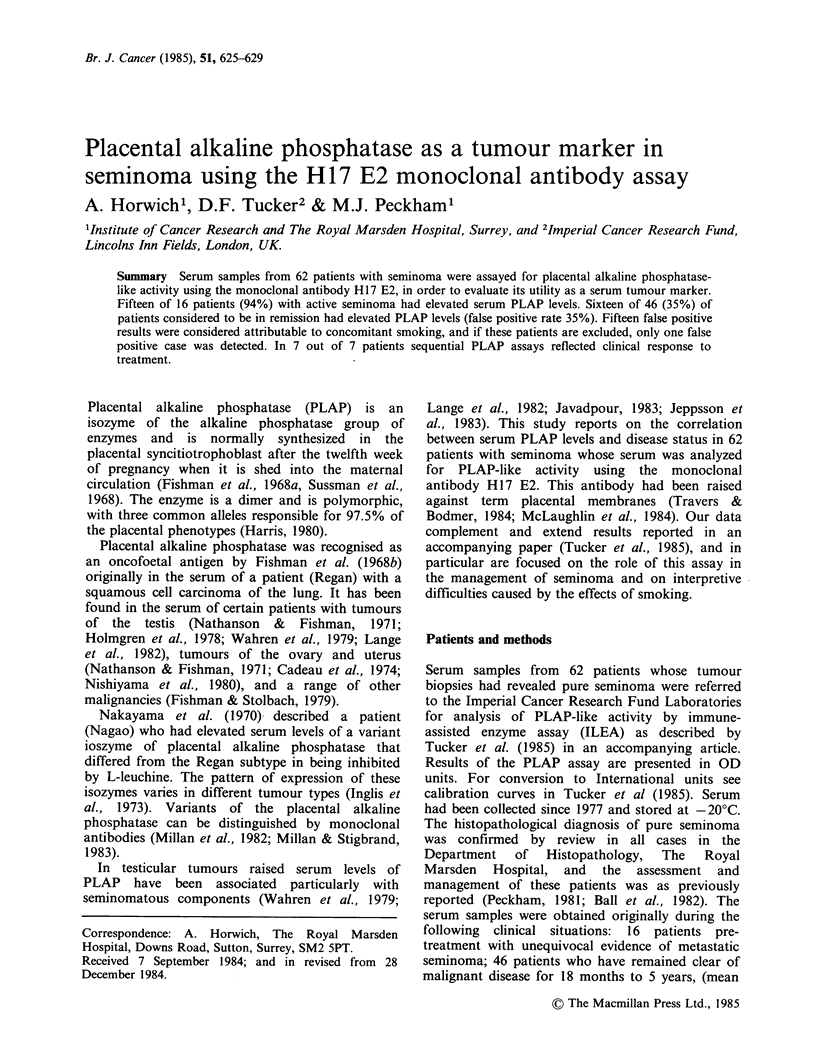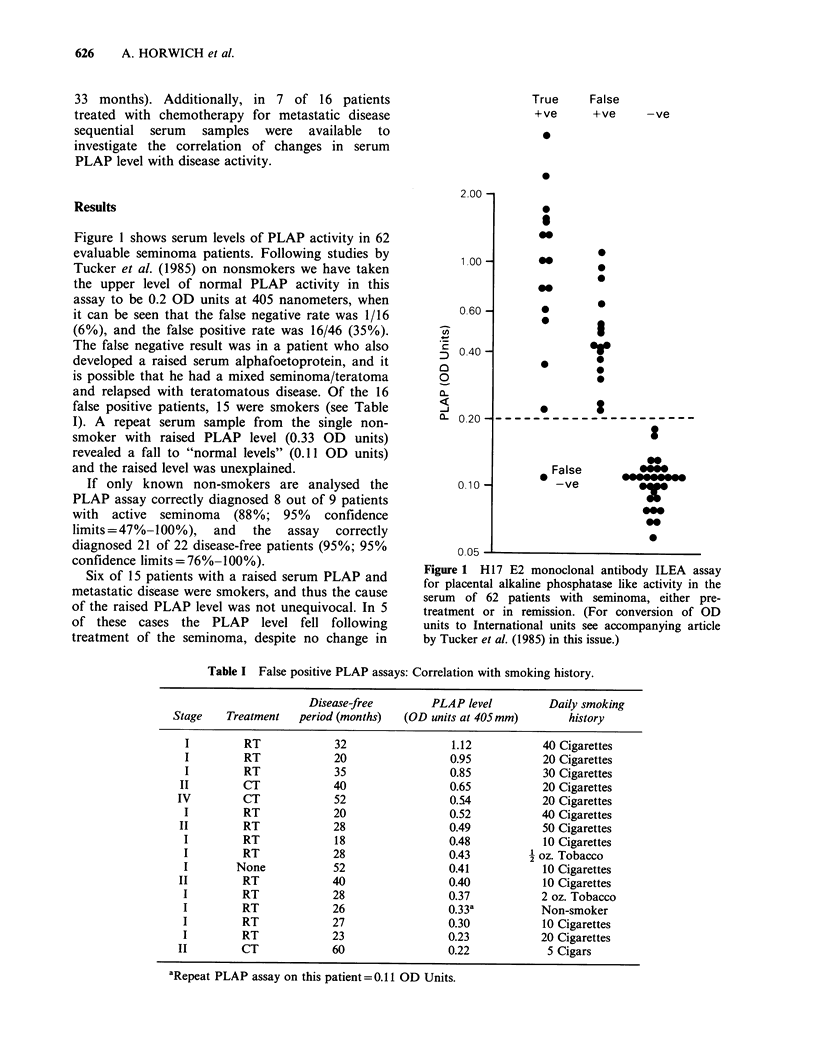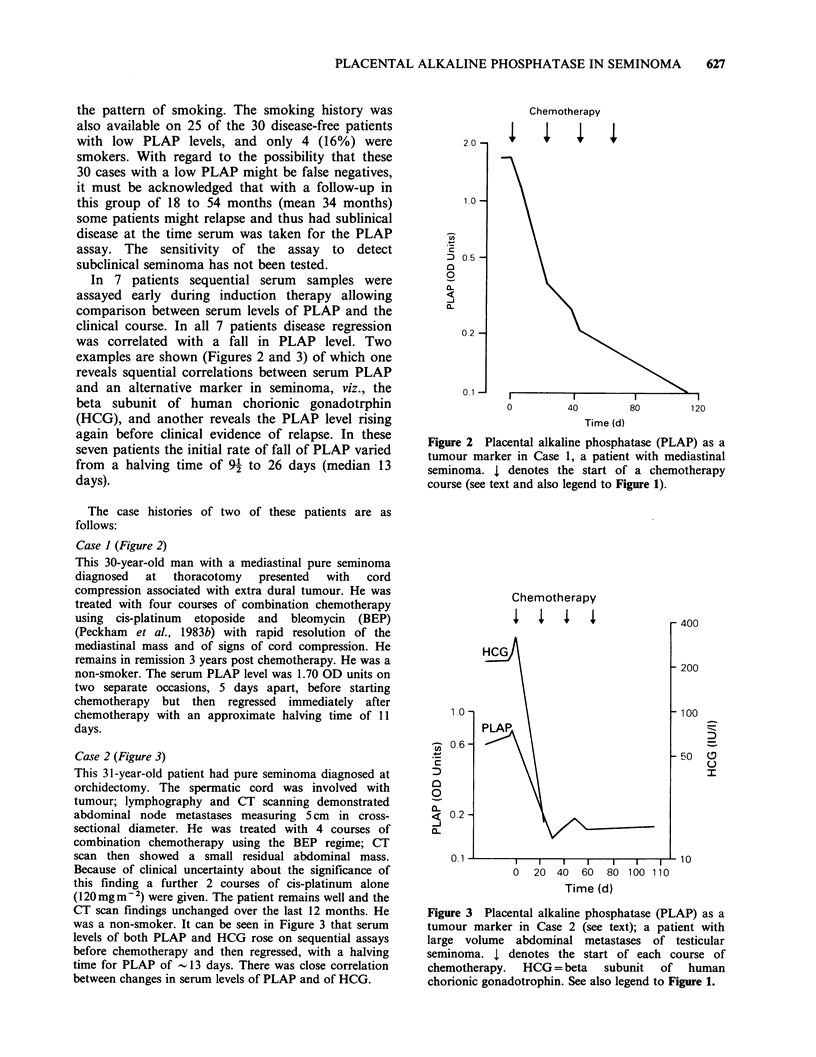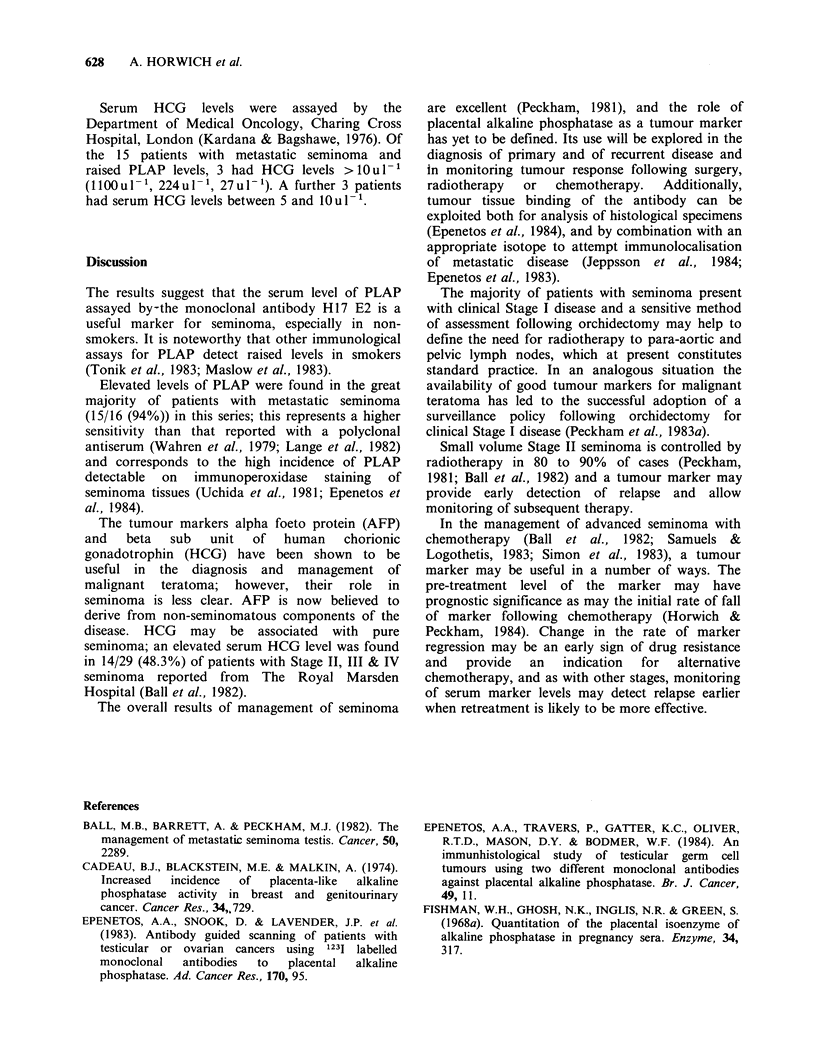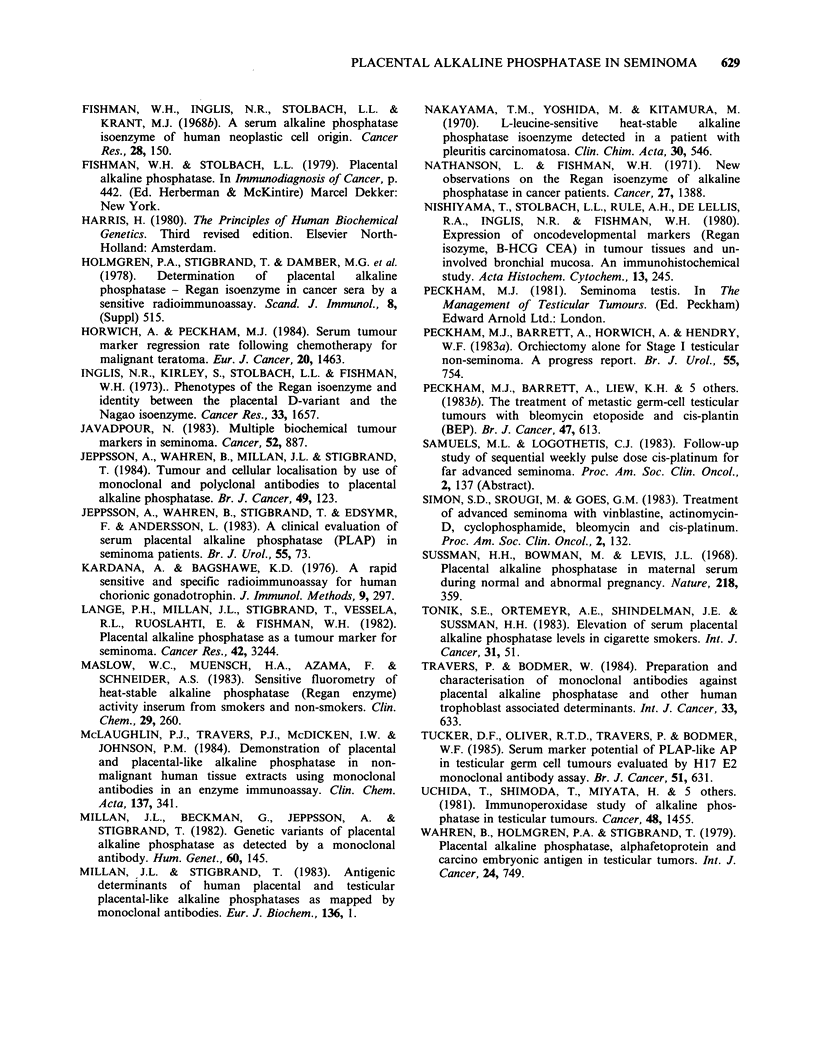# Placental alkaline phosphatase as a tumour marker in seminoma using the H17 E2 monoclonal antibody assay.

**DOI:** 10.1038/bjc.1985.94

**Published:** 1985-05

**Authors:** A. Horwich, D. F. Tucker, M. J. Peckham

## Abstract

Serum samples from 62 patients with seminoma were assayed for placental alkaline phosphatase-like activity using the monoclonal antibody H17 E2, in order to evaluate its utility as a serum tumour marker. Fifteen of 16 patients (94%) with active seminoma had elevated serum PLAP levels. Sixteen of 46 (35%) of patients considered to be in remission had elevated PLAP levels (false positive rate 35%). Fifteen false positive results were considered attributable to concomitant smoking, and if these patients are excluded, only one false positive case was detected. In 7 out of 7 patients sequential PLAP assays reflected clinical response to treatment.


Br. J. Cancer (1985), 51, 625–629

### Placental alkaline phosphatase as a tumour marker in seminoma using the H17 E2 monoclonal antibody assay

A. Horwich[1], D.F. Tucker[2] & M.J. Peckham[1]

[1]*Institute of Cancer Research and The Royal Marsden Hospital, Surrey, and* [2]*Imperial Cancer Research Fund, Lincolns Inn Fields, London, UK.*

**Summary** Serum samples from 62 patients with seminoma were assayed for placental alkaline phosphatase-like activity using the monoclonal antibody H17 E2, in order to evaluate its utility as a serum tumour marker. Fifteen of 16 patients (94%) with active seminoma had elevated serum PLAP levels. Sixteen of 46 (35%) of patients considered to be in remission had elevated PLAP levels (false positive rate 35%). Fifteen false positive results were considered attributable to concomitant smoking, and if these patients are excluded, only one false positive case was detected. In 7 out of 7 patients sequential PLAP assays reflected clinical response to treatment.

Placental alkaline phosphatase (PLAP) is an isozyme of the alkaline phosphatase group of enzymes and is normally synthesized in the placental syncitiotrophoblast after the twelfth week of pregnancy when it is shed into the maternal circulation (Fishman *et al.*, 1968*a*, Sussman *et al.*, 1968). The enzyme is a dimer and is polymorphic, with three common alleles responsible for 97.5% of the placental phenotypes (Harris, 1980).

Placental alkaline phosphatase was recognised as an oncofoetal antigen by Fishman *et al.* (1968*b*) originally in the serum of a patient (Regan) with a squamous cell carcinoma of the lung. It has been found in the serum of certain patients with tumours of the testis (Nathanson & Fishman, 1971; Holmgren *et al.*, 1978; Wahren *et al.*, 1979; Lange *et al.*, 1982), tumours of the ovary and uterus (Nathanson & Fishman, 1971; Cadeau *et al.*, 1974; Nishiyama *et al.*, 1980), and a range of other malignancies (Fishman & Stolbach, 1979).

Nakayama *et al.* (1970) described a patient (Nagao) who had elevated serum levels of a variant ioszyme of placental alkaline phosphatase that differed from the Regan subtype in being inhibited by L-leuchine. The pattern of expression of these isozymes varies in different tumour types (Inglis *et al.*, 1973). Variants of the placental alkaline phosphatase can be distinguished by monoclonal antibodies (Millan *et al.*, 1982; Millan & Stigbrand, 1983).

In testicular tumours raised serum levels of PLAP have been associated particularly with seminomatous components (Wahren *et al.*, 1979; Lange *et al.*, 1982; Javadpour, 1983; Jeppsson *et al.*, 1983). This study reports on the correlation between serum PLAP levels and disease status in 62 patients with seminoma whose serum was analyzed for PLAP-like activity using the monoclonal antibody H17 E2. This antibody had been raised against term placental membranes (Travers & Bodmer, 1984; McLaughlin *et al.*, 1984). Our data complement and extend results reported in an accompanying paper (Tucker *et al.*, 1985), and in particular are focused on the role of this assay in the management of seminoma and on interpretive difficulties caused by the effects of smoking.

### Patients and methods

Serum samples from 62 patients whose tumour biopsies had revealed pure seminoma were referred to the Imperial Cancer Research Fund Laboratories for analysis of PLAP-like activity by immune-assisted enzyme assay (ILEA) as described by Tucker *et al.* (1985) in an accompanying article. Results of the PLAP assay are presented in OD units. For conversion to International units see calibration curves in Tucker *et al* (1985). Serum had been collected since 1977 and stored at −20°C. The histopathological diagnosis of pure seminoma was confirmed by review in all cases in the Department of Histopathology, The Royal Marsden Hospital, and the assessment and management of these patients was as previously reported (Peckham, 1981; Ball *et al.*, 1982). The serum samples were obtained originally during the following clinical situations: 16 patients pre-treatment with unequivocal evidence of metastatic seminoma; 46 patients who have remained clear of malignant disease for 18 months to 5 years, (mean

Correspondence: A. Horwich, The Royal Marsden Hospital, Downs Road, Sutton, Surrey, SM2 5PT.
Received 7 September 1984; and in revised from 28 December 1984.

© The Macmillan Press Ltd., 1985

33 months). Additionally, in 7 of 16 patients treated with chemotherapy for metastatic disease sequential serum samples were available to investigate the correlation of changes in serum PLAP level with disease activity.

### Results

Figure 1 shows serum levels of PLAP activity in 62 evaluable seminoma patients. Following studies by Tucker *et al.* (1985) on nonsmokers we have taken the upper level of normal PLAP activity in this assay to be 0.2 OD units at 405 nanometers, when it can be seen that the false negative rate was 1/16 (6%), and the false positive rate was 16/46 (35%). The false negative result was in a patient who also developed a raised serum alphafoetoprotein, and it is possible that he had a mixed seminoma/teratoma and relapsed with teratomatous disease. Of the 16 false positive patients, 15 were smokers (see Table I). A repeat serum sample from the single non-smoker with raised PLAP level (0.33 OD units) revealed a fall to "normal levels" (0.11 OD units) and the raised level was unexplained.

If only known non-smokers are analysed the PLAP assay correctly diagnosed 8 out of 9 patients with active seminoma (88%; 95% confidence limits = 47%–100%), and the assay correctly diagnosed 21 of 22 disease-free patients (95%; 95% confidence limits = 76%–100%).

Six of 15 patients with a raised serum PLAP and metastatic disease were smokers, and thus the cause of the raised PLAP level was not unequivocal. In 5 of these cases the PLAP level fell following treatment of the seminoma, despite no change in

**Figure 1** H17 E2 monoclonal antibody ILEA assay for placental alkaline phosphatase like activity in the serum of 62 patients with seminoma, either pre-treatment or in remission. (For conversion of OD units to International units see accompanying article by Tucker *et al.* (1985) in this issue.)

**Table I** False positive PLAP assays: Correlation with smoking history.

| *Stage* | *Treatment* | *Disease-free period (months)* | *PLAP level (OD units at 405 mm)* | *Daily smoking history* |
|---|---|---|---|---|
| I | RT | 32 | 1.12 | 40 Cigarettes |
| I | RT | 20 | 0.95 | 20 Cigarettes |
| I | RT | 35 | 0.85 | 30 Cigarettes |
| II | CT | 40 | 0.65 | 20 Cigarettes |
| IV | CT | 52 | 0.54 | 20 Cigarettes |
| I | RT | 20 | 0.52 | 40 Cigarettes |
| II | RT | 28 | 0.49 | 50 Cigarettes |
| I | RT | 18 | 0.48 | 10 Cigarettes |
| I | RT | 28 | 0.43 | ½ oz. Tobacco |
| I | None | 52 | 0.41 | 10 Cigarettes |
| II | RT | 40 | 0.40 | 10 Cigarettes |
| I | RT | 28 | 0.37 | 2 oz. Tobacco |
| I | RT | 26 | 0.33[a] | Non-smoker |
| I | RT | 27 | 0.30 | 10 Cigarettes |
| I | RT | 23 | 0.23 | 20 Cigarettes |
| II | CT | 60 | 0.22 | 5 Cigars |

[a]Repeat PLAP assay on this patient = 0.11 OD Units.

the pattern of smoking. The smoking history was also available on 25 of the 30 disease-free patients with low PLAP levels, and only 4 (16%) were smokers. With regard to the possibility that these 30 cases with a low PLAP might be false negatives, it must be acknowledged that with a follow-up in this group of 18 to 54 months (mean 34 months) some patients might relapse and thus had sublinical disease at the time serum was taken for the PLAP assay. The sensitivity of the assay to detect subclinical seminoma has not been tested.

In 7 patients sequential serum samples were assayed early during induction therapy allowing comparison between serum levels of PLAP and the clinical course. In all 7 patients disease regression was correlated with a fall in PLAP level. Two examples are shown (Figures 2 and 3) of which one reveals squential correlations between serum PLAP and an alternative marker in seminoma, *viz.*, the beta subunit of human chorionic gonadotrphin (HCG), and another reveals the PLAP level rising again before clinical evidence of relapse. In these seven patients the initial rate of fall of PLAP varied from a halving time of 9½ to 26 days (median 13 days).

The case histories of two of these patients are as follows:

*Case 1* (*Figure 2*)

This 30-year-old man with a mediastinal pure seminoma diagnosed at thoracotomy presented with cord compression associated with extra dural tumour. He was treated with four courses of combination chemotherapy using cis-platinum etoposide and bleomycin (BEP) (Peckham *et al.*, 1983*b*) with rapid resolution of the mediastinal mass and of signs of cord compression. He remains in remission 3 years post chemotherapy. He was a non-smoker. The serum PLAP level was 1.70 OD units on two separate occasions, 5 days apart, before starting chemotherapy but then regressed immediately after chemotherapy with an approximate halving time of 11 days.

*Case 2* (*Figure 3*)

This 31-year-old patient had pure seminoma diagnosed at orchidectomy. The spermatic cord was involved with tumour; lymphography and CT scanning demonstrated abdominal node metastases measuring 5 cm in cross-sectional diameter. He was treated with 4 courses of combination chemotherapy using the BEP regime; CT scan then showed a small residual abdominal mass. Because of clinical uncertainty about the significance of this finding a further 2 courses of cis-platinum alone (120 mg m$^{-2}$) were given. The patient remains well and the CT scan findings unchanged over the last 12 months. He was a non-smoker. It can be seen in Figure 3 that serum levels of both PLAP and HCG rose on sequential assays before chemotherapy and then regressed, with a halving time for PLAP of ~13 days. There was close correlation between changes in serum levels of PLAP and of HCG.

**Figure 2** Placental alkaline phosphatase (PLAP) as a tumour marker in Case 1, a patient with mediastinal seminoma. ↓ denotes the start of a chemotherapy course (see text and also legend to **Figure 1**).

**Figure 3** Placental alkaline phosphatase (PLAP) as a tumour marker in Case 2 (see text); a patient with large volume abdominal metastases of testicular seminoma. ↓ denotes the start of each course of chemotherapy. HCG = beta subunit of human chorionic gonadotrophin. See also legend to **Figure 1.**

Serum HCG levels were assayed by the Department of Medical Oncology, Charing Cross Hospital, London (Kardana & Bagshawe, 1976). Of the 15 patients with metastatic seminoma and raised PLAP levels, 3 had HCG levels $>10\,\mathrm{u\,l^{-1}}$ ($1100\,\mathrm{u\,l^{-1}}$, $224\,\mathrm{u\,l^{-1}}$, $27\,\mathrm{u\,l^{-1}}$). A further 3 patients had serum HCG levels between 5 and $10\,\mathrm{u\,l^{-1}}$.

### Discussion

The results suggest that the serum level of PLAP assayed by the monoclonal antibody H17 E2 is a useful marker for seminoma, especially in non-smokers. It is noteworthy that other immunological assays for PLAP detect raised levels in smokers (Tonik *et al.*, 1983; Maslow *et al.*, 1983).

Elevated levels of PLAP were found in the great majority of patients with metastatic seminoma (15/16 (94%)) in this series; this represents a higher sensitivity than that reported with a polyclonal antiserum (Wahren *et al.*, 1979; Lange *et al.*, 1982) and corresponds to the high incidence of PLAP detectable on immunoperoxidase staining of seminoma tissues (Uchida *et al.*, 1981; Epenetos *et al.*, 1984).

The tumour markers alpha foeto protein (AFP) and beta sub unit of human chorionic gonadotrophin (HCG) have been shown to be useful in the diagnosis and management of malignant teratoma; however, their role in seminoma is less clear. AFP is now believed to derive from non-seminomatous components of the disease. HCG may be associated with pure seminoma; an elevated serum HCG level was found in 14/29 (48.3%) of patients with Stage II, III & IV seminoma reported from The Royal Marsden Hospital (Ball *et al.*, 1982).

The overall results of management of seminoma are excellent (Peckham, 1981), and the role of placental alkaline phosphatase as a tumour marker has yet to be defined. Its use will be explored in the diagnosis of primary and of recurrent disease and in monitoring tumour response following surgery, radiotherapy or chemotherapy. Additionally, tumour tissue binding of the antibody can be exploited both for analysis of histological specimens (Epenetos *et al.*, 1984), and by combination with an appropriate isotope to attempt immunolocalisation of metastatic disease (Jeppsson *et al.*, 1984; Epenetos *et al.*, 1983).

The majority of patients with seminoma present with clinical Stage I disease and a sensitive method of assessment following orchidectomy may help to define the need for radiotherapy to para-aortic and pelvic lymph nodes, which at present constitutes standard practice. In an analogous situation the availability of good tumour markers for malignant teratoma has led to the successful adoption of a surveillance policy following orchidectomy for clinical Stage I disease (Peckham *et al.*, 1983*a*).

Small volume Stage II seminoma is controlled by radiotherapy in 80 to 90% of cases (Peckham, 1981; Ball *et al.*, 1982) and a tumour marker may provide early detection of relapse and allow monitoring of subsequent therapy.

In the management of advanced seminoma with chemotherapy (Ball *et al.*, 1982; Samuels & Logothetis, 1983; Simon *et al.*, 1983), a tumour marker may be useful in a number of ways. The pre-treatment level of the marker may have prognostic significance as may the initial rate of fall of marker following chemotherapy (Horwich & Peckham, 1984). Change in the rate of marker regression may be an early sign of drug resistance and provide an indication for alternative chemotherapy, and as with other stages, monitoring of serum marker levels may detect relapse earlier when retreatment is likely to be more effective.

### References

BALL, M.B., BARRETT, A. & PECKHAM, M.J. (1982). The management of metastatic seminoma testis. *Cancer*, **50**, 2289.

CADEAU, B.J., BLACKSTEIN, M.E. & MALKIN, A. (1974). Increased incidence of placenta-like alkaline phosphatase activity in breast and genitourinary cancer. *Cancer Res.*, **34**,,729.

EPENETOS, A.A., SNOOK, D. & LAVENDER, J.P. *et al.* (1983). Antibody guided scanning of patients with testicular or ovarian cancers using $^{123}$I labelled monoclonal antibodies to placental alkaline phosphatase. *Ad. Cancer Res.*, **170**, 95.

EPENETOS, A.A., TRAVERS, P., GATTER, K.C., OLIVER, R.T.D., MASON, D.Y. & BODMER, W.F. (1984). An immunhistological study of testicular germ cell tumours using two different monoclonal antibodies against placental alkaline phosphatase. *Br. J. Cancer*, **49**, 11.

FISHMAN, W.H., GHOSH, N.K., INGLIS, N.R. & GREEN, S. (1968*a*). Quantitation of the placental isoenzyme of alkaline phosphatase in pregnancy sera. *Enzyme*, **34**, 317.

FISHMAN, W.H., INGLIS, N.R., STOLBACH, L.L. & KRANT, M.J. (1968*b*). A serum alkaline phosphatase isoenzyme of human neoplastic cell origin. *Cancer Res.*, **28**, 150.

FISHMAN, W.H. & STOLBACH, L.L. (1979). Placental alkaline phosphatase. In *Immunodiagnosis of Cancer*, p. 442. (Ed. Herberman & McKintire) Marcel Dekker: New York.

HARRIS, H. (1980). *The Principles of Human Biochemical Genetics*. Third revised edition. Elsevier North-Holland: Amsterdam.

HOLMGREN, P.A., STIGBRAND, T. & DAMBER, M.G. *et al.* (1978). Determination of placental alkaline phosphatase – Regan isoenzyme in cancer sera by a sensitive radioimmunoassay. *Scand. J. Immunol.*, **8**, (Suppl) 515.

HORWICH, A. & PECKHAM, M.J. (1984). Serum tumour marker regression rate following chemotherapy for malignant teratoma. *Eur. J. Cancer*, **20**, 1463.

INGLIS, N.R., KIRLEY, S., STOLBACH, L.L. & FISHMAN, W.H. (1973).. Phenotypes of the Regan isoenzyme and identity between the placental D-variant and the Nagao isoenzyme. *Cancer Res.*, **33**, 1657.

JAVADPOUR, N. (1983). Multiple biochemical tumour markers in seminoma. *Cancer*, **52**, 887.

JEPPSSON, A., WAHREN, B., MILLAN, J.L. & STIGBRAND, T. (1984). Tumour and cellular localisation by use of monoclonal and polyclonal antibodies to placental alkaline phosphatase. *Br. J. Cancer*, **49**, 123.

JEPPSSON, A., WAHREN, B., STIGBRAND, T. & EDSYMR, F. & ANDERSSON, L. (1983). A clinical evaluation of serum placental alkaline phosphatase (PLAP) in seminoma patients. *Br. J. Urol.*, **55**, 73.

KARDANA, A. & BAGSHAWE, K.D. (1976). A rapid sensitive and specific radioimmunoassay for human chorionic gonadotrophin. *J. Immunol. Methods*, **9**, 297.

LANGE, P.H., MILLAN, J.L., STIGBRAND, T., VESSELA, R.L., RUOSLAHTI, E. & FISHMAN, W.H. (1982). Placental alkaline phosphatase as a tumour marker for seminoma. *Cancer Res.*, **42**, 3244.

MASLOW, W.C., MUENSCH, H.A., AZAMA, F. & SCHNEIDER, A.S. (1983). Sensitive fluorometry of heat-stable alkaline phosphatase (Regan enzyme) activity inserum from smokers and non-smokers. *Clin. Chem.*, **29**, 260.

McLAUGHLIN, P.J., TRAVERS, P.J., McDICKEN, I.W. & JOHNSON, P.M. (1984). Demonstration of placental and placental-like alkaline phosphatase in non-malignant human tissue extracts using monoclonal antibodies in an enzyme immunoassay. *Clin. Chem. Acta*, **137**, 341.

MILLAN, J.L., BECKMAN, G., JEPPSSON, A. & STIGBRAND, T. (1982). Genetic variants of placental alkaline phosphatase as detected by a monoclonal antibody. *Hum. Genet.*, **60**, 145.

MILLAN, J.L. & STIGBRAND, T. (1983). Antigenic determinants of human placental and testicular placental-like alkaline phosphatases as mapped by monoclonal antibodies. *Eur. J. Biochem.*, **136**, 1.

NAKAYAMA, T.M., YOSHIDA, M. & KITAMURA, M. (1970). L-leucine-sensitive heat-stable alkaline phosphatase isoenzyme detected in a patient with pleuritis carcinomatosa. *Clin. Chim. Acta*, **30**, 546.

NATHANSON, L. & FISHMAN, W.H. (1971). New observations on the Regan isoenzyme of alkaline phosphatase in cancer patients. *Cancer*, **27**, 1388.

NISHIYAMA, T., STOLBACH, L.L., RULE, A.H., DE LELLIS, R.A., INGLIS, N.R. & FISHMAN, W.H. (1980). Expression of oncodevelopmental markers (Regan isozyme, B-HCG CEA) in tumour tissues and uninvolved bronchial mucosa. An immunohistochemical study. *Acta Histochem. Cytochem.*, **13**, 245.

PECKHAM, M.J. (1981). Seminoma testis. In *The Management of Testicular Tumours*. (Ed. Peckham) Edward Arnold Ltd.: London.

PECKHAM, M.J., BARRETT, A., HORWICH, A. & HENDRY, W.F. (1983*a*). Orchiectomy alone for Stage I testicular non-seminoma. A progress report. *Br. J. Urol.*, **55**, 754.

PECKHAM, M.J., BARRETT, A., LIEW, K.H. & 5 others. (1983*b*). The treatment of metastic germ-cell testicular tumours with bleomycin etoposide and cis-plantin (BEP). *Br. J. Cancer*, **47**, 613.

SAMUELS, M.L. & LOGOTHETIS, C.J. (1983). Follow-up study of sequential weekly pulse dose cis-platinum for far advanced seminoma. *Proc. Am. Soc. Clin. Oncol.*, **2**, 137 (Abstract).

SIMON, S.D., SROUGI, M. & GOES, G.M. (1983). Treatment of advanced seminoma with vinblastine, actinomycin-D, cyclophosphamide, bleomycin and cis-platinum. *Proc. Am. Soc. Clin. Oncol.*, **2**, 132.

SUSSMAN, H.H., BOWMAN, M. & LEVIS, J.L. (1968). Placental alkaline phosphatase in maternal serum during normal and abnormal pregnancy. *Nature*, **218**, 359.

TONIK, S.E., ORTEMEYR, A.E., SHINDELMAN, J.E. & SUSSMAN, H.H. (1983). Elevation of serum placental alkaline phosphatase levels in cigarette smokers. *Int. J. Cancer*, **31**, 51.

TRAVERS, P. & BODMER, W. (1984). Preparation and characterisation of monoclonal antibodies against placental alkaline phosphatase and other human trophoblast associated determinants. *Int. J. Cancer*, **33**, 633.

TUCKER, D.F., OLIVER, R.T.D., TRAVERS, P. & BODMER, W.F. (1985). Serum marker potential of PLAP-like AP in testicular germ cell tumours evaluated by H17 E2 monoclonal antibody assay. *Br. J. Cancer*, **51**, 631.

UCHIDA, T., SHIMODA, T., MIYATA, H. & 5 others. (1981). Immunoperoxidase study of alkaline phosphatase in testicular tumours. *Cancer*, **48**, 1455.

WAHREN, B., HOLMGREN, P.A. & STIGBRAND, T. (1979). Placental alkaline phosphatase, alphafetoprotein and carcino embryonic antigen in testicular tumors. *Int. J. Cancer*, **24**, 749.